# Veterinarians Experience Animal Welfare Control Work as Stressful

**DOI:** 10.3389/fvets.2020.00077

**Published:** 2020-02-19

**Authors:** Sofia Väärikkälä, Laura Hänninen, Mari Nevas

**Affiliations:** ^1^Department of Food Hygiene and Environmental Health, Faculty of Veterinary Medicine, University of Helsinki, Helsinki, Finland; ^2^Research Centre for Animal Welfare, Faculty of Veterinary Medicine, University of Helsinki, Helsinki, Finland; ^3^Department of Production Animal Medicine, Faculty of Veterinary Medicine, University of Helsinki, Helsinki, Finland

**Keywords:** animal welfare control, job satisfaction, official veterinarian, overtime work, work-related stress

## Abstract

The aim of the study was to evaluate the job satisfaction of official veterinarians working in the field of animal welfare control and identify both positive features and challenges of their work. An electronic questionnaire was designed to evaluate job satisfaction. The questionnaire was responded to by 73 of the 98 Finnish official veterinarians working in the field of animal welfare control. The Spearman's rank correlation coefficient was used to evaluate the relation between stress and different work-related factors. More than half of the respondents reported work-related stress or fatigue. Threatening situations, disturbed work–private life balance and a high amount of overtime work were found to be frequent underlying causes of stress. Fieldwork, especially when working alone, was perceived as the most challenging part of the work. Of the respondents, three out of four performed animal welfare inspections mainly alone. Although the respondents reported getting additional help to perform an inspection most of the times they needed it, a wish to work in a pair was highlighted. The results of the present study indicate that official veterinarians often experience work-related stress and fatigue. By testing interventions shown to be beneficial, such as providing adequate support within the work community, decreasing the workload and enabling inspections to be done in pairs, job satisfaction could be improved.

## Introduction

A wide range of legislative provisions concerning animal welfare have been established over the last 30 years in the European Union (EU). The Member States are obligated to implement official controls in order to monitor and verify that animal welfare standards are complied with (1). The demands concerning controls are explicit but the Member States have the freedom to decide how to implement them. An overview of how official controls are organized in the Member States is provided by the European Commission (2).

In Finland, the implementation of animal welfare control is organized at four administrative levels (3): the Ministry of Agriculture and Forestry, the Finnish Food Authority, the Regional State Administrative Agencies, and the local authorities. Provincial veterinary officers working in the regional agencies are responsible for the animal welfare inspections of farmed animals and animal transport requested by the EU. The local authorities, mainly municipal veterinarians, but also the police, and health inspectors, control the compliance with animal welfare standards within the territory of the municipality. In 2017, ~15 provincial veterinary officers and 60 municipal veterinarians worked full-time in the field of animal welfare control. In addition to the authorities, animal welfare inspectors authorized by the Regional State Administrative Agencies may perform animal welfare inspections. The veterinary officer for meat inspection controls compliance with animal welfare standards at slaughterhouses, and the veterinary officer for border control controls compliance at border crossings, exits, and veterinary border control points.

According to the Finnish Animal Welfare Act (3) the animal welfare inspections may be performed based on a suspicion of non-compliance with the animal welfare standards, and on regular intervals on certain animal premises (e.g. in circuses, zoos, permanent, and traveling animal shows, and places with professional or other large-scale keeping of pet and hobby animals). In addition, a sample of farms are inspected every year as requested by the EU legislation (1). The provincial veterinary officers focus mostly on the farm inspections, whereas, the municipal veterinarians perform mainly suspicion-based inspections and work with the whole range of animal species. In 2017, the official veterinarians performed 377 sample-based farm-animal inspections, of which 78% concerned cattle farms. In addition, 6,448 suspicion-based inspections were performed, of which ~60% concerned companion animals (4).

Official veterinarians work in a demanding environment–animals must be protected from unnecessary pain and suffering, yet they have to respect the basic rights of a person to own property, including animals, and to earn a living, including through livestock production. Lepistö (5) called this dilemma “the conflict of basic rights in the field of environmental health and food control”. Also, Tannenbaum (6) argued that veterinarians face difficult ethical questions as servants of both animal, and human interests, which may conflict. In addition, individuals with different backgrounds perceive animals, and their welfare differently (7), and this may cause conflicts as not all are satisfied with the work of the animal welfare authorities who may not require more than the minimum standards laid down in legislation. In addition, animal owners may not be satisfied with unequal treatment if standards are open to various interpretations (8). Good communication skills are required as controls contain a lot of face-to-face interaction (9), and reactions during controls can be hostile and sometimes even aggressive (10).

There are several studies on the well-being of veterinarians and it has been shown that the rates of suicidal behavior [see for review (11)], stress (12–14), and mental health problems (12, 15) are elevated. Causes of veterinarian stress include long work hours, conflicting client relations (16), interference with the work–home balance (17), low income, high debt (14), ethical conflicts, and moral distress (18).

The previous studies have mainly concentrated on clinical veterinarian practitioners, and according to the authors' knowledge there are no studies on the well-being of veterinarians working mainly in the field of animal welfare control. To bridge the gap, this study focuses on the special characteristics of these veterinarians with the aim of evaluating their working conditions and job satisfaction.

## Materials and Methods

An electronic questionnaire was developed together with the Finnish Veterinary Association to acquire information on working conditions of the Finnish official veterinarians and their well-being at work. The questionnaire included a cover letter in which the purpose of the study, voluntary participation, and confidentiality were explained. In addition, it was indicated that by completing the questionnaire a respondent consents to participate the study. The questionnaire was sent by the Finnish Veterinary Association after obtaining from the Regional State Administrative Agencies an email address list of the official veterinarians currently working, or that have recently worked, in the field of animal welfare control. A hyperlink to the questionnaire was sent by email to 98 recipients during autumn 2017. It was open for 3 weeks and a reminder was sent 10 days before the closing of the questionnaire. The Finnish Veterinary Association collected the responses electronically and deleted all identifying data before sending the data to the authors. No follow-up study to investigate the reasons for not responding was performed.

Ethical review was not applied for as the study did not meet any of the criteria defined by the Finnish Advisory Board on Research Integrity Ethical (19).

The questionnaire included closed, multiple choice, and open-ended questions covering the following topics: (1) background information including work experience, current position, and work content, (2) positive features and challenges of the work, (3) job satisfaction and negative side effects of the work, (4) experiencing work-related stress, (5) support from and cooperation with different partners, and (6) use of enforcement measures and educational needs (Supplementary Material).

### Statistical Analysis

The respondents were categorized based on their work history as a veterinarian, an official veterinarian, and on their current position. The Spearman's rank correlation coefficient was used to evaluate the strength, and direction of a relationship between different variables. Only correlations with coefficients over 0.30 are reported. The Mann–Whitney *U*-test and the Kruskal–Wallis H test for ordinal variables, and the Fisher's exact test for nominal variables were used to evaluate the differences between the variables. Non-parametric analysis was chosen as the Kolmogorov-Smirnov-test of normality showed that the data were not normally distributed (*p* < 0.05 for all).

To deepen the information gained through the closed and multiple-choice questions, some of the open-ended questions were analyzed by using content analysis (20), in which the data were coded, and categorized to identify common themes.

The “Don't know” answers were categorized as “missing,” and ambiguous answers in open questions that could not be interpreted were excluded from the analysis. Statistical significance was accepted at a confidence level of 95% (*p* < 0.05). The data were analyzed using SPSS statistical software (IBM SPSS Statistics 22.0, NY, USA).

## Results

### Background Information and Work Content

A total of 73 responses were given (response rate 74%). All the respondents worked in the field of animal welfare control. In addition, the job description of most respondents included animal health and disease control and/ or by-product control. Some of the respondents also worked in the field of food control. The respondents' background information including work history and current position, and parameters related to their work are presented in Table 1.

**Table 1:** Background information of the respondents and parameters related to their work.

			**Job satisfaction**				**Frequency of stress**			
**Variable**	**Category**	**Total *n* (%)**	**Good**	**Variable**	**Bad**	***p*-value^a^**	**Rarely or never**	**Sometimes**	**Weekly or more often**	***p*-value^a^**
Worked as veterinarian										
	<3 years	16 (22)	10 (63)	6 (38)	0 (0)	0.27	1 (6)	7 (44)	8 (50)	0.84
	3–5 years	14 (19)	6 (43)	6 (43)	2 (14)		2 (14)	4 (29)	8 (57)	
	6–15 years	14 (19)	5 (36)	7 (50)	2 (14)		1 (7)	6 (43)	7 (50)	
	>15 years	29 (40)	15 (52)	12 (41)	2 (7)		4 (14)	10 (35)	15 (52)	
Worked as official veterinarian										
	<1 year	13 (18)	8 (62)	4 (31)	1 (8)	0.46	2 (15)	6 (46)	5 (39)	0.42
	1–3 years	24 (33)	11 (46)	10 (42)	3 (13)		0 (0)	9 (38)	15 (63)	
	4–7 years	26 (36)	13 (50)	11 (42)	2 (8)		5 (19)	8 (31)	13 (50)	
	>7 years	10 (14)	4 (40)	6 (60)	0 (0)		1 (10)	4 (40)	5 (50)	
Current position										
	Municipal veterinarian	63 (86)	30 (48)	27 (43)	6 (10)	0.33	8 (13)	22 (35)	33 (52)	0.57
	Provincial veterinary officer	10 (14)	6 (60)	4 (40)	0 (0)		0 (0)	5 (50)	5 (50)	
Job description										
	Animal welfare control	73 (100)	NA	NA	NA	NA	NA	NA	NA	NA
	Animal health and disease control	66 (90)	NA	NA	NA		NA	NA	NA	
	By-product control	50 (69)	NA	NA	NA		NA	NA	NA	
	Food control	28 (38)	NA	NA	NA		NA	NA	NA	
Animal welfare inspections per week										
	1–5 inspections	50 (69)	24 (48)	21 (42)	5 (10)	0.50	4 (8)	18 (36)	28 (56)	0.42
	6–10 inspections	20 (27)	11 (55)	8 (40)	1 (5)		4 (20)	8 (40)	8 (40)	
	>10 inspections	3 (4)	1 (33)	2 (67)	0 (0)		0 (0)	1 (33)	2 (67)	
Animal welfare inspections performed										
	Alone	55 (75)	24 (44)	25 (46)	6 (11)	0.08	6 (11)	20 (36)	29 (53)	0.56
	With a pair	18 (25)	12 (67)	6 (30)	0 (0)		2 (11)	7 (39)	9 (50)	
Possibility to get somebody to come with to perform inspection										
	Always	31 (42)	19 (61)	10 (32)	2 (7)	0.07	6 (19)	9 (29)	16 (52)	0.16
	Often	18 (25)	7 (39)	7 (39)	4 (22)		0 (0)	5 (28)	13 (72)	
	Sometimes	7 (10)	2 (29)	5 (71)	0 (0)		0 (0)	4 (57)	3 (43)	
	Only when prearranged	17 (23)	8 (47)	9 (53)	0 (0)		2 (12)	9 (53)	6 (35)	
Work phone open only during office hours										
	Yes	52 (71)	29 (56)	23 (44)	0 (0)	0.93	4 (7)	21 (40)	27 (52)	0.86
	No	21 (29)	10 (48)	8 (38)	3 (14)		4 (19)	6 (29)	11 (52)	
Acute animal welfare cases outside office hours in past 12 months										
	None	43 (60)	24 (56)	18 (42)	1 (2)	0.10	5 (12)	20 (47)	18 (42)	0.31
	Once	11 (15)	6 (55)	4 (36)	1 (9)		2 (18)	2 (18)	7 (64)	
	2–4 times	12 (17)	3 (25)	7 (58)	2 (17)		0 (0)	5 (42)	7 (58)	
	>4 times	6 (8)	2 (33)	2 (33)	2 (33)		1 (17)	0 (0)	5 (83)	
Support from superior and work community										
	Always	19 (26)	13 (68)	5 (26)	1 (5)	0.002^*^	4 (21)	8 (42)	7 (37)	0.14
	Often	27 (37)	16 (59)	7 (26)	4 (15)		1 (4)	11 (41)	15 (56)	
	Sometimes	21 (29)	3 (14)	17 (81)	1 (5)		1 (5)	6 (29)	14 (67)	
	Never	1 (1)	1 (100)	0 (0)	0 (0)		0 (0)	1 (100)	0 (0)	
	If asked for	5 (7)	3 (60)	2 (40)	0 (0)		2 (40)	1 (20)	2 (40)	
Possibility to work from home										
	Yes	51 (70)	24 (47)	23 (45)	4 (8)	0.40	4 (8)	19 (37)	28 (55)	0.34
	No	22 (30)	12 (55)	8 (36)	2 (9)		4 (18)	8 (36)	10 (46)	
Working overtime										
	Daily	11 (15)	4 (36)	5 (46)	2 (18)	0.29	0 (0)	0 (0)	11 (100)	0.008^*^
	Weekly	27 (37)	14 (52)	11 (41)	2 (7)		1 (4)	11 (41)	15 (56)	
	Few times per month	19 (26)	11 (58)	7 (37)	1 (5)		3 (16)	11 (58)	5 (26)	
	More rarely	15 (21)	7 (47)	8 (53)	0 (0)		4 (27)	5 (33)	6 (40)	
Commitment to work										
	Too low	0 (0)	0 (0)	0 (0)	0 (0)	0.01^*^	0 (0)	0 (0)	0 (0)	<0.001^*^
	Suitable	49 (67)	29 (59)	17 (35)	3 (6)		8 (16)	23 (47)	18 (37)	
	Too high	24 (33)	7 (29)	14 (58)	3 (13)		0 (0)	4 (17)	20 (83)	
Meaningfulness of work										
	Very meaningfulness	20 (28)	18 (90)	2 (10)	0 (0)	<0.001^*^	7 (35)	9 (45)	4 (20)	<0.001^*^
	Somewhat meaningfulness	33 (47)	16 (48)	17 (52)	0 (0)		1 (3)	15 (46)	17 (52)	
	Somewhat meaningless	13 (18)	0 (0)	12 (92)	1 (8)		0 (0)	2 (15)	11 (85)	
	Very meaningless	5 (7)	0 (0)	0 (0)	5 (100)		0 (0)	0 (0)	5 (100)	
Loneliness at work										
	Always	4 (7)	0 (0)	4 (100)	0 (0)	0.001^*^	0 (0)	2 (50)	2 (50)	<0.001^*^
	Often	29 (40)	10 (35)	16 (55)	3 (10)		0 (0)	7 (24)	22 (76)	
	Sometimes	33 (46)	19 (57)	11 (33)	3 (9)		5 (15)	15 (46)	13 (39)	
	Never	6 (8)	6 (100)	0 (0)	0 (0)		3 (50)	2 (33)	1 (17)	
Sleeping disorders because of work										
	Always	1 (1)	0 (0)	1 (100)	0 (0)	0.04^*^	0 (0)	0 (0)	1 (100)	<0.001^*^
	Often	12 (16)	3 (25)	7 (58)	2 (17)		0 (0)	2 (17)	10 (83)	
	Sometimes	51 (70)	26 (51)	21 (41)	4 (8)		3 (6)	21 (41)	27 (53)	
	Never	9 (12)	7 (78)	2 (22)	0 (0)		5 (56)	4 (44)	0 (0)	
Disturbed work-private life balance										
	Lot	8 (11)	1 (13)	6 (75)	1 (13)	<0.001^*^	0 (0)	0 (0)	8 (100)	<0.001^*^
	Somewhat	37 (51)	14 (38)	18 (49)	5 (14)		0 (0)	10 (27)	27 (73)	
	Little	22 (30)	16 (73)	6 (27)	0 (0)		4 (18)	15 (69)	3 (14)	
	None	6 (8)	5 (83)	1 (17)	0 (0)		4 (67)	2 (33)	0 (0)	
Threatening situations at work in the past 12 months										
	Yes	64 (88)	30 (47)	28 (44)	6 (9)	0.10	4 (6)	24 (38)	36 (56)	0.009^*^
	No	9 (12)	6 (67)	3 (33)	0 (0)		4 (44)	3 (33)	2 (22)	

a *Mann-Whitney U-test or Kruskal-Wallis T-test were used to test the difference between the categories*.

* *Significant difference (p < 0.05). NA, not applicable*.

### Challenges and Negative Side Effects of Work

The respondents ranked fieldwork, including inspections and sampling, as the most challenging part of their work (Table 2). This was explained by working alone, communication problems, and too high a workload. Nearly all of the respondents (93%; 63/68) perceived working alone as inconvenient. The most common reasons for this were: compromised safety at work, challenges of making adequate observations alone, and perceived insecurity of own legal protection.

**Table 2:** Most challenging elements of the work perceived by official veterinarians.

**Element**	***n* (%)**
Fieldwork	23 (32)
Interpretation of legislation	11 (15)
Paperwork	10 (14)
Reporting	9 (12)
Other	12 (18)

More than half of the respondents estimated that they experience work-related stress or fatigue at least weekly (Table 1). The high frequency of stress was associated with threatening situations, such as death threats, assaults, and disturbance of domestic peace, high frequency of overtime work, too high a commitment to work, disturbed work–private life balance, and the inconvenience of working alone (Table 3). The respondents who had encountered threatening situations at work more often experienced sleeping disorders and suffered from loneliness than those respondents who had not encountered threatening situations (*p* < 0.01 for both). The most used means of coping in the threatening situations were “discussing,” “fleeing from the situation,” and “calling the police.”

**Table 3:** Spearman correlation between work-related factors and stress among official veterinarians.

**Work-related factor**	**Stress**	
	***r***	***p***
Threatening situations	0.37	0.01
Overtime work	0.44	<0.001
Commitment to work	0.47	<0.001
Work-private life balance	−0.71	<0.001
Inconvenience of working alone	0.35	0.01

The more the respondents were working overtime and participating in animal welfare control outside office hours, the more often they perceived the commitment to work as too high (*r* = 0.55 and *r* = 0.31, respectively, *p* < 0.01 for both). When the respondents perceived their commitment to work as too high, they also more often experienced sleeping disorders and disturbed work–private life balance (*r* = 0.34 and *r* = 0.50, respectively, *p* < 0.01 for both).

Neither length of veterinary career nor current position seemed to influence the respondents' experience of the stress or other negative side effects.

### Positive Features of Work

Nearly half of the respondents reported having good job satisfaction and nearly every third respondent perceived their work as very meaningful (Table 1). There was a strong relationship between job satisfaction and sense of the meaningfulness of the work (*r* = 0.75, *p* < 0.001). The impact of the work, i.e., the possibility to help animals, was ranked as the best element of the work (Table 4).

**Table 4:** Best elements of the work perceived by official veterinarians.

**Element**	***n* (%)**
Impact of the work, i.e., possibility of helping animals	42 (63)
Regular working hours but still flexibility and independence	20 (30)
Work community	13 (19)
Versatility of work	12 (18)
People (both work community and clients) met at work	10 (15)

The respondents reported receiving support mostly in the form of the exchange of views with colleagues, guidance from the Regional State Administrative Agencies, and support given by a supervisor. The more easily the respondents received support, the more often they perceived themselves to be suitably committed to their work (*r* = 0.32, *p* = 0.01), the less they suffered from loneliness (*r* = 0.41, *p* = 0.001), and sleeping disorders (*r* = 0.31, *p* = 0.01), and the better they perceived their job satisfaction to be (*r* = 0.36, *p* = 0.003). The most desired forms of support were possibility to work in a pair, supervision of work and legal advice. Two thirds of the respondents (48/73) expressed their wish to use enforcement measures together with a colleague at least in difficult cases. The respondents listed legislative education, education on animal welfare and husbandry, and training in interaction skills as the most important educational needs.

The respondents perceived cooperation with different partners to be mostly well-functioning (Table 5). If the respondents perceived cooperation with the work community, the supervisor and the police to be well-functioning, they also more often perceived their work to be meaningful (*r* = 0.34, *r* = 0.35, and *r* = 0.31, respectively, *p* < 0.02 for all). Cooperation with the work community was perceived to be better functioning and the respondents felt less lonely when it was easy to obtain a partner for performing an inspection (*r* = 0.50 and *r* = 0.46, respectively, *p* < 0.001). The respondents reported that they performed inspections most often with an official veterinarian working either as a practitioner or in the field of animal welfare control, the police or a health inspector. The respondents who performed animal welfare inspections in a pair perceived their work to be more meaningful than those working alone (*p* = 0.01).

**Table 5:** Perceptions of official veterinarians on how well the cooperation with different parties function.

**Partner**	**Very well *n* (%)**	**Well *n* (%)**	**Neither well nor badly *n* (%)**	**Badly *n* (%)**	**Very badly *n* (%)**	**I don't know *n* (%)**
Work community	22 (31)	39 (54)	8 (11)	2 (3)	0 (0)	1 (1)
Superior	30 (41)	30 (41)	8 (11)	5 (6)	0 (0)	0 (0)
Regional State Administrative Agency^a^	22 (34)	23 (36)	11 (17)	7 (11)	0 (0)	1 (1)
Finnish Food Safety Authority	6 (8)	25 (34)	30 (41)	7 (10)	1 (1)	4 (6)
Police	17 (23)	33 (45)	16 (22)	5 (7)	1 (1)	1 (1)
Prosecutor	10 (14)	24 (33)	13 (18)	6 (8)	0 (0)	20 (27)
Social services	4 (5)	24 (33)	17 (24)	3 (4)	1 (1)	23 (32)
Child protection services	3 (4)	22 (31)	14 (19)	4 (6)	1 (1)	28 (39)

a *Only the respondents working at the local level estimated how well the cooperation functions with the Regional State Administrative Agency*.

## Discussion

The Finnish official veterinarians working in the field of animal welfare control perceive their work as meaningful because they can influence the welfare of animals, but at the same time, they often experience work-related stress and fatigue. The underlying causes of stress were identified as threatening situations, such as death threats, assaults, and disturbance of domestic peace, high frequency of overtime work, disturbed work–private life balance, and the inconvenience of working alone. Long working hours have also previously been recognized as a stress factor among veterinarians (13, 16). Although no direct relationship between work support and work–private life balance was found in this study, it was shown that support helps to decrease sleeping disorders. It is crucial that official veterinarians have the possibility to process work problems at work instead of worrying about them alone or with a family member at home. It has been suggested that to bring work–private life into balance, job control, and supervisor support should be available (21).

Most of the veterinarians performed animal welfare inspections alone, most likely due to resource and financial reasons, though they perceived it inconvenient and wished to work in pairs. If the usual inspection tasks, such as observing, taking measurements, and photos, writing notes, and at the same time communicating with the client(s), were divided between two persons, it would decrease the workload of an individual veterinarian and safeguard the veterinarians', and animal owner's legal protection. The veterinarians were worried about their safety at work, and their feeling of insecurity is justified as most of them had already encountered a threatening situation at work. Clients' physical assaults are significant stressors also for human health care workers (22). The impact of working in pairs to the efficacy of animal welfare control should be further studied, i.e., whether the use of enforcement measures is enhanced and the instructions are better followed when given by two persons rather than one.

Working alone, communication problems and too high a workload were reasons why fieldwork was experienced as the most challenging part of the work of official veterinarians. Performing inspections at the homes of clients and animal facilities even against the client's will and enforcing animal welfare legislation exposes the veterinarians to challenging interactions with the clients. The impact of a client's gender on the interaction was not examined in this study; however, we previously showed that female Finnish farmers perceive animal welfare inspections more positively than male farmers and that a client's positive attitude toward inspection is associated with better interaction (9). The level of experienced stress did not differ between the provincial veterinary officers and the municipal veterinarians, though they perform different types of inspections and have different target species. More research is needed to evaluate the possible differences between the inspections concerning certain animal species and the stress experience of official veterinarians.

Veterinarians may also face social and health-related human factors as these are often behind animal welfare problems (23, 24). Official veterinarians may not have adequate professional qualifications to get through challenging client interactions when the clients also have serious personal problems (25). The responding official Finnish veterinarians required more training in interaction skills. This was quite opposite to the Irish governmental veterinarians who did not want to have more education in this area (25). It may be beneficial to add more communication training for veterinarians in order to enhance veterinarian–client interaction (26). The importance of good communication skills was highlighted in this study, as the most important act in the threatening situations was reported to be discussing.

In this study, most official veterinarians wished to use the enforcement measures together with a colleague at least in difficult cases. The enforcement measures most often applied in animal welfare control are orders to perform correct actions within a specific time period and prohibitions for continuing or repeating an illegal procedure (4). Further, official veterinarians may also take immediate action to ensure the welfare of an animal, for example by taking animals away from their owners (3). Enforcement measures may conflict with the basic rights of the animal owners, such as their freedom to conduct a business, and they may have long-lasting, for example economic, effects. Thus, the correct and impartial use of enforcement measures is important. It is commonly understood that veterinarians should not assume a passive role when facing serious welfare matters (27); however, Finnish veterinarians have been criticized for being passive in animal welfare cases by Koskela (28). One reason for passivity might be a veterinarian's uncertainty in making difficult decisions alone, resulting in repetitive inspections before an animal welfare case is solved. Another reason leading to unfinished cases might be a fear of facing, and a desire to avoid, a client after a threatening situation (29). Although veterinary education provides a strong base, an extensive knowledge of enforcement tools is a prerequisite for them to be decisive (10). Kettunen et al. (30) have suggested that allocated and practical training on administrative procedures should be provided to strengthen the skills, and confidence of officials in using enforcement measures. Plausible explanation for the wish of the official veterinarians to undertake enforcement measures with a colleague is that they want to share responsibility and, thus, avoid the hostility directed to a single veterinarian. The municipal veterinarians use the enforcement measures independently, while provincial veterinary officers use them on the behalf of an organization. The decisions should be made under the name of an organization or two persons rather than one person.

The support received from the supervisor and the work community was shown to be very important for official veterinarians. When performing inspections alone and making decisions independently on issues open for interpretation, the role of the working community as provider of support becomes highly relevant. The importance of support and the opportunity to meet and reflect on the experiences with colleagues has been recognized also by Anneberg et al. (31) and Devitt et al. (25). The importance of good cooperation with the police is important as the police are not only relevant in the process of animal welfare crime investigation but also for providing assistance during animal welfare inspections.

One of the strengths of this study is high response rate (74%). The current position of the responding veterinarians corresponded well with the overall distribution of official veterinarians in Finland. No gender or demographic information were collected as this information would have disclosed the identity of some respondents. The distribution by gender of all veterinarians in Finland is ~1:3, female veterinarians being in the majority. The confidentiality was also secured by letting the respondents respond anonymously, and by deleting all possible identifying data on the responses of the open-ended questions before sending the data to the authors.

This study provides novel, valuable information about the well-being of official veterinarians whose job content differ considerably from veterinarians conducting clinical veterinary practice; enforcing animal welfare legislation may result in difficult interaction with a client, and even to threatening situations. By providing adequate support within the work community, decreasing the workload, and enabling inspections to be done in pairs, job satisfaction of official veterinarians could be improved.

There are, however, also weaknesses of the study. First, only the frequency of stress was inquired about, not the severity. Second, the number of respondents in some groups, such as groups categorized based on the current position, was significantly smaller than in others, making a comprehensive comparison between the groups not possible. Thirdly, bias caused by social desirability is also possible and strong dissatisfaction with work might have been a motivation to respond.

## Conclusions

Animal welfare control work is often experienced as stressful. Having the possibility to work in a pair, adequate resources to minimize overtime work, and a well-managed, supportive work community were shown to be beneficial to the workplace well-being of official veterinarians. The findings support the testing of these interventions when aiming at improving working conditions. To be prepared for threatening situations, veterinarians should receive more training in interaction skills and cooperate well with the police.

## Data Availability Statement

The datasets generated for this study are available on request to the corresponding author.

## Ethics Statement

Ethical review and approval was not required for the study on human participants in accordance with the local legislation and institutional requirements. Written informed consent was not provided because the participants were informed that by returning the completed questionnaire they would be considered to have given consent to participate in this study.

## Author Contributions

SV analyzed the data, interpreted the results, and wrote the first draft of the manuscript. LH helped with drafting the manuscript and supervised. MN participated in the drafting of the questionnaire, supervised, and helped draft the manuscript.

### Conflict of Interest

The authors declare that the research was conducted in the absence of any commercial or financial relationships that could be construed as a potential conflict of interest.
