# Supplementary material for: Veterinarians Experience Animal Welfare Control Work as Stressful
Source: Front Vet Sci. 2020 Feb 19;7:77. doi: 10.3389/fvets.2020.00077 (PMC7042310; doi:10.3389/fvets.2020.00077)
Supplement: Supplementary file 1 [file Table_1.DOCX]

Supplementary Material. The relevant questions of the questionnaire aimed at official veterinarians

# Background information and work content

## What does your job description include (you may choose more than one option)? Please estimate the proportion of time you use for each.

- Animal welfare control
- Animal health and disease control
- Food control
- Fish plant control
- Milk hygiene control
- By-product control
- Other, what?

## How did you end up being an official veterinarian (you may choose more than one option)?

- I found the job significant
- I did not want to work as a practitioner
- I wanted to have a job without emergency duty
- A vacancy opened at the right moment
- A temporary vacancy opened at the right moment
- Other, what?

## How many years have you worked as a veterinarian?

- < 3 years
- 3–5 years
- 6–15 years
- > 15 years

## How many years have you worked as an official veterinarian in the field of animal welfare?

- < 1 year
- 1–3 years
- 4–6 years
- > 7 years

## What is your current position?

- Municipal
- Regional State Administrative Agency

## How many animal welfare inspections do you perform on average per week?

- 1–5
- 6–10
- > 10

## How many times have you participated in animal welfare cases outside office hours in the past 12 months?

- None
- Once
- 2–4 times
- > 4 times
- I don’t know

## How do you perceive your work commitment?

- Too little
- Suitable
- Too high

## How often do you work overtime?

- Daily
- Weekly
- Few times per month
- More rarely

## Do you have the possibility to work from home?

- Yes
- No

## Which inspections do you perform alone (you may choose more than one option)?

- Animal welfare control
- Animal health and disease control
- Food control
- Fish plant control
- Milk hygiene control
- By-product control
- Other, what?

## How big a proportion of animal welfare inspections do you perform alone?

- < 10%
- 10–30%
- 31–50%
- 51–80%
- > 80%

## Are you able to get somebody to come with you to perform an inspection?

- Never
- Sometimes
- Often
- Always
- Only when prearranged
- I don’t know

## Who do you get to come with you?

- Practitioner
- Another official veterinarian
- Police
- Health inspector
- Animal welfare counsellor
- Some other, who?

## How do you use your work phone outside office hours (you may choose more than one option)?

- Phone is off
- I use voicemail
- I answer colleagues
- I answer the police
- I always keep the phone on

# Positive features and challenges of the work

## What are the best elements of your work?

## What are the most challenging elements of your work (please select from the list) and why?

- Fieldwork
- Paperwork
- Interpretation of legislation
- Reporting
- Other, what?

## Do you perceive working alone as inconvenient?

- Never
- Sometimes
- Often
- Always

## Why do you perceive working alone as inconvenient?

## Do you feel lonely at work?

- Never
- Sometimes
- Often
- Always
- I don’t know

## What kind of threatening situations have you encountered at work?

- I have been threatened to be killed
- I have been threatened in another way
- I have been assaulted
- My phone has been interfered with
- My home has been targeted
- Other, what?

## How have you managed in a threatening situation (you may choose more than one option)?

- By discussing
- By defending myself
- By using a weapon
- By using something that I had in my hands or could reach
- By calling the police
- By fleeing from the situation
- Other, what?

# Job satisfaction and negative side effects of work

## How do you perceive your job satisfaction?

- Very good
- Good
- Variable
- Bad
- Very bad

## How meaningful do you perceive your work?

- Very meaningful
- Somewhat meaningful
- Somewhat unmeaningful
- Very unmeaningful
- Neither meaningful nor unmeaningful

## Does the work affect your sleep?

- Never
- Sometimes
- Often
- Always

## Does the work negatively affect your private life?

- No
- Only a little
- Somewhat
- A lot

# Experiencing work-related stress

## Do you experience work-related stress or fatigue?

- Never
- Seldom
- Sometimes
- Weekly
- Daily
- I don’t know

# Support and cooperation

## Do you get support from your superior and work community?

- Never
- Sometimes
- Often
- Always
- Yes, if I ask for it

## What kind of support do you receive?

## What kind of support do you wish to have?

## How well does the cooperation with your superior, the Regional State Administrative Agency, the Food Safety Authority, the police, the prosecutor, social workers and child protection workers work?

- Very well
- Well
- Neither well nor badly
- Badly
- Very badly
- I don’t know

# Use of enforcement measures and educational needs

## How do you perceive the use of enforcement measures?

- Challenging
- I can use enforcement measures well
- I need more training to use them correctly
- I don’t perceive the use as difficult
- I don’t know

## How would you like to use the enforcement measures?

- By myself
- The Regional Administrative Agency should use them on behalf of local veterinarians
- Together with a veterinarian working as a practitioner
- Together with another official veterinarian
- Other, what?

## What kind of training would you like to have (more than one option can be chosen)?

- Training in interaction skills
- Education in animal welfare and husbandry
- Education in animal diseases
- Education in legislation
- Other, what?
